# Pediatric Maxillary Strut Anatomy: A CT-Based Analysis of Morphometry, Shape, and Pneumatization

**DOI:** 10.3390/jcm15166186

**Published:** 2026-08-10

**Authors:** Burak Bahadır, Nur Hürsoy, Fatma Betül Saylak, Eda Aslanbaba Bahadır, Orhan Beger

**Affiliations:** 1 Department of Neurosurgery, Ankara Bilkent City Research and Training Hospital, University of Health Sciences, TR-06800 Ankara, Türkiye; 2 Department of Radiology, Faculty of Medicine, Recep Tayyip Erdoğan University, TR-53020 Rize, Türkiye; nur.hursoy@erdogan.edu.tr; 3 Department of Neurosurgery, Çankırı State Hospital, TR-18100 Çankırı, Türkiye; fbetul.saylak@gmail.com; 4 Department of Neurology, Ankara Gölbaşı Şehit Ahmet Özsoy State Hospital, TR-06830 Ankara, Türkiye; edaaslanbaba@gmail.com; 5 Department of Anatomy, Faculty of Medicine, Gaziantep University, TR-27310 Gaziantep, Türkiye; obeger@gmail.com

**Keywords:** maxillary strut, pediatric anatomy, computed tomography, morphometric analysis, pneumatization, skull base

## Abstract

**Objective:** To characterize age- and sex-related differences in maxillary strut (MS) morphometry, shape, and pneumatization in children using computed tomography (CT). **Methods:** Cranial CT examinations from 180 children aged 1–18 years were retrospectively evaluated. After one participant with a rudimentary unilateral MS was excluded, the primary bilateral complete-case analyses included 179 children and 358 MSs (179 right and 179 left). The anteroposterior diameter (MS-AP), area (MS-A), and mediolateral diameter (MS-ML) were measured bilaterally. Each side was classified as trapezoidal or hourglass-shaped, yielding bilateral trapezoidal, bilateral hourglass-shaped, or asymmetric participant-level patterns. **Results:** All morphometric parameters were higher in males than in females (MS-AP and MS-ML, *p* < 0.001; MS-A, *p* = 0.008), with no significant right–left differences. All parameters differed across chronological age strata and age groups (all *p* < 0.001). Age-by-sex interactions were significant for MS-AP (*p* < 0.001) and MS-A (*p* = 0.001), but not for MS-ML (*p* = 0.257). Bilateral trapezoidal, bilateral hourglass-shaped, and asymmetric patterns occurred in 40.8%, 30.2%, and 29.1% of participants, respectively. Shape pattern was associated with sex (*p* = 0.003), but not with age group (*p* = 0.359), and the right–left shape distributions did not differ significantly (*p* = 0.070). Pneumatization was unilateral in 4 out of 179 participants (2.2%; exact 95% confidence interval: 0.6–5.6%). **Conclusions:** Pediatric MS dimensions vary with age and sex, while shape shows a sex-related distribution and pneumatization is uncommon. These CT-based findings provide baseline anatomical data on pediatric MS development and may inform future radiological and surgical research.

## 1. Introduction

The maxillary strut (MS) is a distinct bony projection arising from the greater wing of the sphenoid that contributes to the division of the primitive foramen lacerum anterius into the foramen rotundum (FR) and superior orbital fissure (SOF), forming the osseous boundary between them. Medially, the MS is related to the lateral wall of the sphenoid sinus and the medial portion of the pterygoid process, whereas laterally, it is bordered by the temporal lobe dura [1]. Its location between the FR and SOF, in close relationship with the maxillary nerve and adjacent neurovascular structures, makes it a recognizable anatomical landmark in endoscopic and transcranial skull-base approaches. Previous anatomical and surgical studies have examined its relationship to operative corridors involving the pterygopalatine fossa, orbital apex, cavernous sinus, and middle cranial fossa [1,2,3,4,5]. However, dedicated morphometric evidence regarding the MS remains sparse, with only limited data available from adult studies.

The anatomy of the FR–SOF region may also be relevant in children. Robins et al. [6] treated a 15-year-old patient with a cavernous sinus tumor through a subtemporal extradural approach involving pterional osteoplastic craniotomy, interdural dissection at the SOF, and drilling of the middle fossa floor to improve access to the lesion. Lena et al. [7] achieved total resection of an intracranial maxillary division of the trigeminal nerve (V2) choristoma involving the FR in a 12-year-old girl through a left pterional extradural approach after enlargement of the FR using a high-speed drill. Pediatric cranial and skull-base anatomy, diagnostic and therapeutic considerations, and the biological behavior of related pathologies may differ substantially from those in adults [8,9]. Moreover, continued skull-base maturation throughout childhood and adolescence limits the direct extrapolation of adult morphometric findings to pediatric patients. Systematic pediatric computed tomography (CT) data are therefore needed to define developmental anatomical patterns, facilitate the interpretation of variation, and provide a foundation for future clinical investigations.

To our knowledge, the present study is the first dedicated CT-based evaluation of MS anatomy in children. Accordingly, this study aimed to comprehensively characterize MS morphometry, shape, and pneumatization across the pediatric age range and assess the reproducibility of these evaluations.

## 2. Materials and Methods

### 2.1. Ethical Considerations

The study protocol was reviewed and approved by the No. 2 Medical Research Scientific and Ethical Evaluation Board (TABED) of Ankara Bilkent City Hospital, Ministry of Health, Republic of Türkiye (approval date: 15 October 2025; approval no.: TABED 2-25-1270). Owing to the retrospective design and the exclusive use of anonymized imaging and clinical data, the ethics committee waived the requirement for individual informed consent.

### 2.2. Study Design

This retrospective CT-based study included pediatric patients aged 1–18 years who underwent cranial CT examinations between January 2020 and February 2025. Demographic information, clinical indications, relevant medical history, and radiological findings were reviewed to determine eligibility. The electronic hospital archive was reviewed in reverse chronological order, beginning with the examinations performed in February 2025. Patients were evaluated according to the predefined inclusion and exclusion criteria and categorized into 18 prespecified age strata. To standardize age assignment, the 1- to 17-year age strata were defined using a four-month eligibility window extending from two months before to two months after the corresponding birthday. For example, the 1-year age stratum included children aged 10–14 months. For the 18-year age stratum, only the two-month interval preceding the 18th birthday was used; participants were eligible from 17 years 10 months up to and including the date of their 18th birthday, and no post-birthday extension was applied. Within each age-and-sex stratum, eligibility and image quality were assessed during the reverse-chronological archive review. Five examinations were selected, with priority given to those closest to the corresponding birthday when sufficient eligible cases were available; the full eligibility window was used when necessary, particularly in younger strata with fewer eligible examinations. This procedure was repeated for all 18 age strata, resulting in an age- and sex-balanced cohort of 180 children. The sampling framework was based on previous pediatric CT morphometric studies [10,11].

### 2.3. Power Analysis

Because the cohort size was fixed by the stratified sampling design, no a priori sample-size calculation was used to determine enrollment. A sensitivity power analysis was performed using G*Power version 3.1.9.7 (Heinrich Heine University Düsseldorf, Düsseldorf, Germany) based on the final complete-case sample of 179 participants. The reference analysis was the omnibus comparison of participant-level bilateral mean MS-AP values across the five pediatric age groups using a fixed-effects one-way analysis of variance (ANOVA). With five groups, α = 0.05, and 80% power, the minimum detectable effect size was Cohen’s f = 0.262, where Cohen’s f represents the standardized between-group variability relative to the within-group variability. For f = 0.30, the estimated power was 0.906. This calculation applied only to the reference morphometric analysis and did not establish adequate power for subgroup comparisons involving the four observed cases of pneumatization.

### 2.4. Inclusion and Exclusion Criteria

Patients were eligible if they were within one of the prespecified age-stratum windows, had undergone cranial CT for a clinical indication such as minor trauma, a fall, or headache, and had CT images of sufficient quality for evaluation of the MS. Clinical records and CT images were also reviewed to confirm the absence of a craniofacial condition affecting the region of interest. 

Patients were excluded if they were outside the prespecified age-stratum windows; had documented systemic corticosteroid use or received other medications known to affect bone metabolism; had a genetic disorder, systemic disease, or congenital anomaly that could affect bone development; had undergone previous craniofacial surgery; had a fracture, tumor, or other osseous lesion involving the skull base or region of interest; or had CT images of insufficient quality for evaluation of the MS.

### 2.5. Study Cohort

This retrospective CT investigation included 180 children with a mean age of 9.5 ± 5.2 years, comprising 90 boys and 90 girls.

### 2.6. Computed Tomography Protocol

CT imaging was performed with a 64-detector scanner (VCT XTe LightSpeed; General Electric, Milwaukee, WI, USA). The acquisition protocol included automatic tube current modulation of 150–250 mAs, a 512 × 512 image matrix, tube voltage ranging from 80 to 120 kV, a pitch of 1, a rotation time of 0.35 s, a reconstruction interval of 0.625 mm, a detector thickness of 0.625 mm, variable field of view ranging from 150 to 300 mm, a collimation setting of 40 mm (64 × 0.625 mm), reconstruction with an edge-enhancing Bone Plus kernel, and evaluation using high-contrast pediatric bone window settings (Window Width: 3000–4000 Hounsfield units [HU], Window Level: 600–750 HU). A protocol specifically adapted for pediatric patients was applied throughout the study. Following axial image acquisition, three-dimensional reconstructions were generated in all cases, and all datasets were processed using an Advantage Workstation (GE).

### 2.7. Maxillary Strut Morphometric Measurements

**Figure 1:**
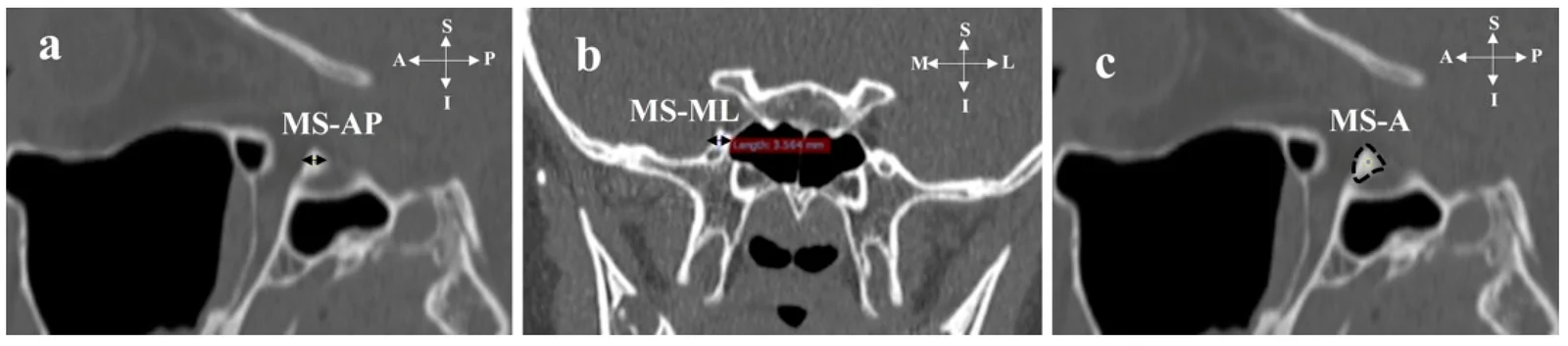
Computed tomography-based measurement of maxillary strut morphometric parameters. (**a**) MS-AP, the maximum anteroposterior dimension measured on a sagittal reformatted image; (**b**) MS-ML, the maximum mediolateral thickness measured on a coronal reformatted image (Length: 3.564 mm); and (**c**) MS-A, the cross-sectional area delineated on an oblique sagittal image perpendicular to the long axis of the strut. MS, maxillary strut; MS-AP, maxillary strut anteroposterior diameter; MS-ML, maxillary strut mediolateral diameter; MS-A, maxillary strut cross-sectional area; A, anterior; P, posterior; S, superior; I, inferior; M, medial; L, lateral.

Three parameters were assessed to characterize the anatomy of the MS (Figure 1): (a) the anteroposterior diameter (MS-AP), (b) the mediolateral diameter (MS-ML), and (c) the cross-sectional area (MS-A). The MS was first identified on three-dimensional (3D) CT reconstructions as the bony bridge between the SOF and the FR, and its location was subsequently verified on coronal and sagittal reformatted images. All measurements were obtained on sectional images, and the corresponding measurement planes are shown in Figure 1. 

For each side, the contiguous sectional images were systematically reviewed. MS-AP was recorded as the greatest anteroposterior osseous dimension of the strut across the reviewed images, whereas MS-ML was recorded as its greatest transverse bony thickness. In very thin segments, where the thickness was approximately 1 mm, caliper markers on 3D reconstructions were used only as a visual aid to estimate the osseous margins, and the definitive measurements were based on sectional imaging. For MS-A, the MS was first localized on the coronal images. Using the multiplanar reconstruction tool, an oblique sagittal plane was adjusted separately for each side to lie perpendicular to the long axis of the strut. The adjacent reformatted slices were then reviewed using the standardized bone-window settings described above, and MS-A was measured on the slice demonstrating complete cortical delineation with the least continuity with adjacent bony structures. The same plane-construction procedure was applied independently by both observers.

### 2.8. Shape of the Maxillary Strut

**Figure 2:**
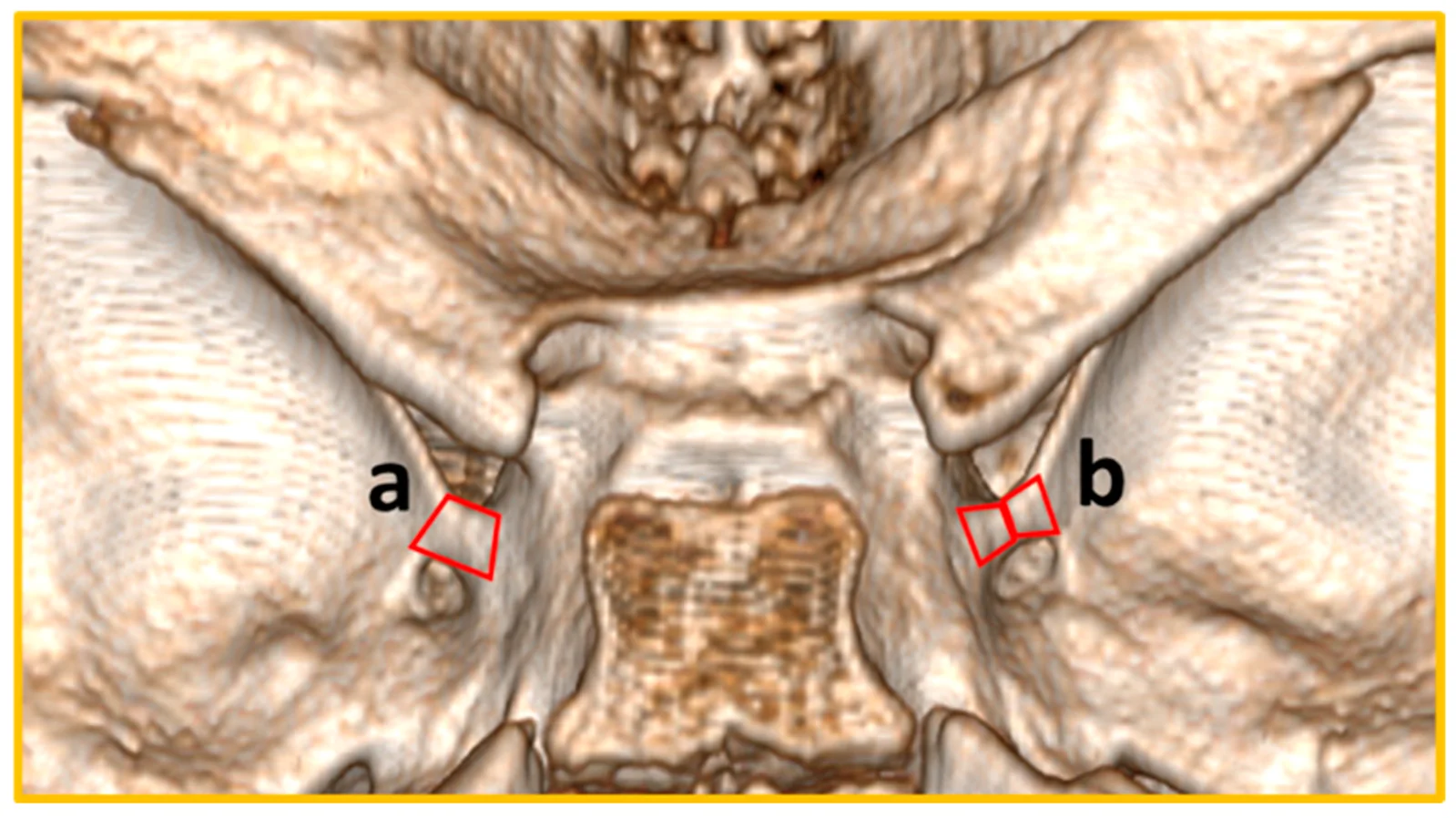
Representative 3D CT appearances of maxillary strut shape classifications: (a) trapezoidal type demonstrating a relatively regular four-sided configuration, and (b) hourglass-shaped type demonstrating a relatively slender configuration with narrowing of the central portion.

The shape of the MS was evaluated on 3D CT reconstructions based on its external osseous contour. Each evaluable MS was classified as either trapezoidal or hourglass-shaped (Figure 2). The trapezoidal type was defined as a relatively regular four-sided configuration resembling a trapezoid without marked central narrowing. The hourglass-shaped type was defined as a configuration in which the central portion of the MS was visibly narrower than both ends.

### 2.9. Pneumatization of the Maxillary Strut

**Figure 3:**
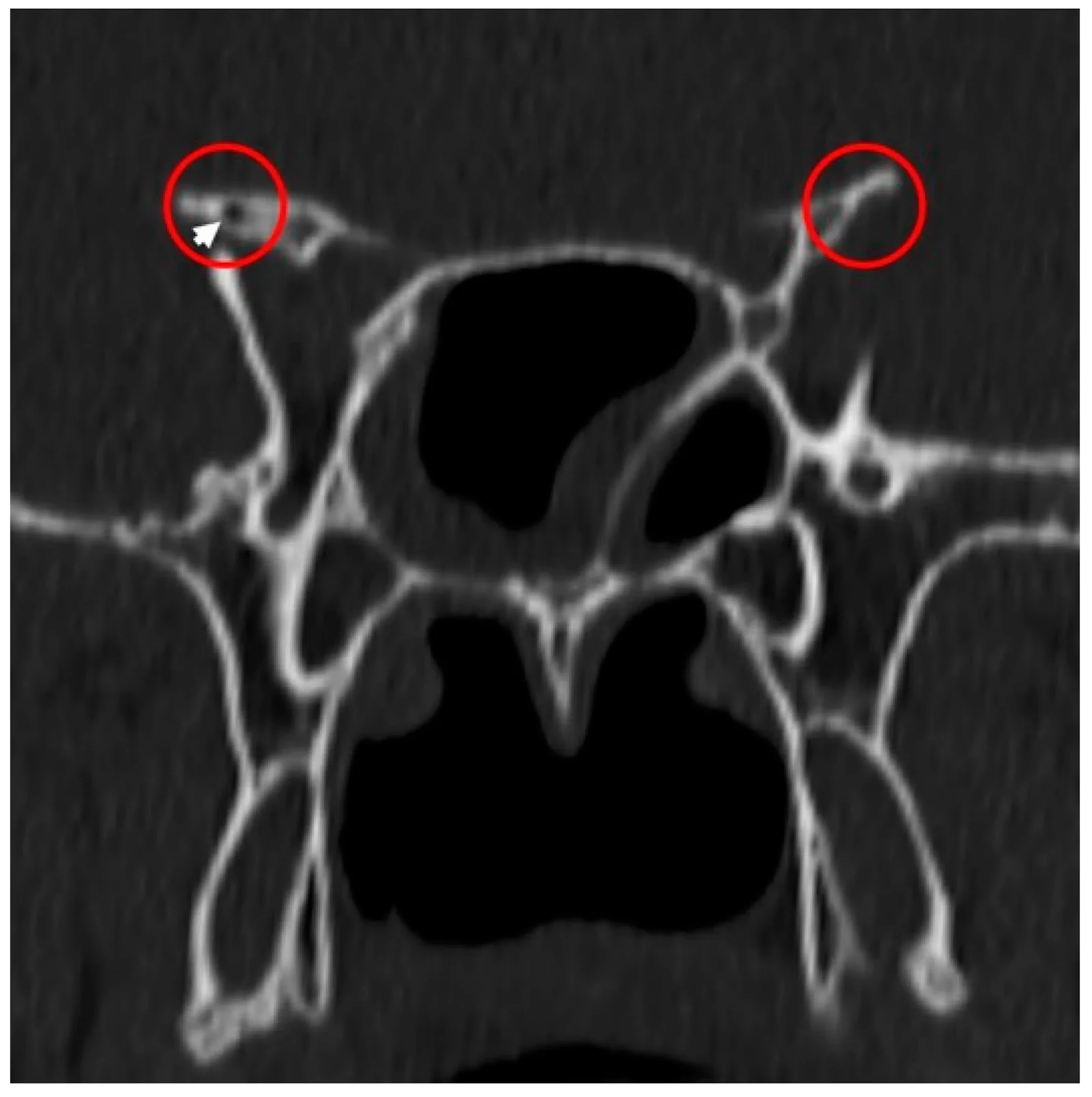
Representative coronal CT image demonstrating maxillary strut pneumatization. The white arrow indicates a visible air-containing cell within the maxillary strut. The red circles indicate the location of the maxillary struts.

Presence or absence of MS pneumatization was noted using sectional imaging (Figure 3). Pneumatization was defined as the presence of a visible air-containing cavity within the MS on sectional CT images.

### 2.10. Statistical Analysis

All statistical analyses were performed using IBM SPSS Statistics for Windows, version 25.0 (IBM Corp., Armonk, NY, USA). Before the measurements, a study-specific data file listing the included examinations was prepared. During the initial assessment, all three morphometric parameters were measured bilaterally and independently by a radiologist (N.H.) with approximately eight years of post-specialty experience and a neurosurgeon (B.B.) with approximately six years of post-specialty experience. Each observer evaluated the examinations independently and recorded each parameter once without access to the other observer’s measurements. The assessment order was not separately randomized. For each parameter, the mean of the measurements recorded by the two observers was used in the statistical analyses. Interobserver reliability was evaluated using a two-way random-effects, absolute-agreement, average-measures intraclass correlation coefficient [ICC(2,2)] with 95% confidence intervals.

During the same initial assessment, the primary shape and pneumatization classifications were determined by consensus between N.H. and B.B. using the predefined criteria described above. These categorical classifications were subsequently reassessed independently by another neurosurgeon (F.B.S.), who applied the same criteria without access to the primary consensus classifications. Agreement between the primary consensus classifications and the blinded independent reassessment was evaluated using Cohen’s kappa coefficient and percentage agreement. The 95% confidence intervals for kappa were obtained from 50,000 participant-level bootstrap resamples. Because N.H. and B.B. accessed the CT examinations through the electronic hospital archive using patient record numbers, they were not blinded to participant age or sex. F.B.S. was blinded specifically to the primary shape and pneumatization classifications.

Continuous variables were summarized as mean ± standard deviation and categorical variables as number and percentage. Distributional assumptions were evaluated primarily using quantile–quantile (Q–Q) plots of the model residuals, supplemented by Shapiro–Wilk tests. Homogeneity of variance in the sex- and age-group comparisons was assessed using Levene’s test. Regression diagnostics included residual-versus-fitted and Q–Q plots, the Koenker–Breusch–Pagan test for heteroscedasticity, externally studentized residuals, and Cook’s distance. Observations with an absolute externally studentized residual greater than 3 were flagged as potential outliers, whereas Cook’s distance greater than 1 was considered evidence of substantial influence. No observation was excluded solely on the basis of these diagnostics; instead, the models were re-estimated without the flagged observations to assess the robustness of the findings. Participant-level morphometric values were calculated as the mean of the right- and left-sided measurements. Because the primary participant-level morphometric and categorical analyses were based on complete bilateral data, and side comparisons required complete bilateral pairs, the participant with a rudimentary right-sided MS was excluded from the primary complete-case analyses. The rudimentary MS was not assigned to either the trapezoidal or hourglass-shaped category, and no missing values were imputed. As a sensitivity analysis, all unpaired participant-level morphometric analyses were repeated using the non-rudimentary contralateral measurements as this participant’s values (*n* = 180); paired side comparisons and categorical analyses remained based on the 179 participants with complete bilateral data. 

Sex-based comparisons were performed using Welch’s *t*-test for MS-AP and MS-A and the independent-samples *t*-test for MS-ML. Right–left comparisons were conducted using the paired-samples *t*-test. Mean differences were reported with 95% confidence intervals, together with Hedges’ g for sex comparisons and Cohen’s dz for paired side comparisons. Age-related morphometric changes were evaluated using regression models incorporating linear and quadratic age terms, sex, and age-by-sex interactions. Age was centered at the sample mean, and final models were selected according to term significance, adjusted coefficient of determination (R^2^), and residual diagnostics. For presentation, the fitted regression equations were back-transformed and expressed using chronological age in years. Because heteroscedasticity was identified in the MS-AP and MS-A models, heteroscedasticity-consistent type 3 (HC3) standard errors were used for these models; conventional ordinary least-squares standard errors were retained for MS-ML. For MS-AP, the linear and quadratic age-by-sex interaction terms were evaluated jointly using an HC3-robust Wald test. Morphometric differences across the 18 chronological age strata were assessed using Welch’s analysis of variance for MS-AP and MS-A and one-way analysis of variance for MS-ML; no pairwise comparisons were performed among the 18 individual ages. Participants were also classified into five age groups: Group 1, 1–2 years; Group 2, 3–5 years; Group 3, 6–9 years; Group 4, 10–13 years; and Group 5, 14–18 years [12]. Differences among these groups were assessed using Welch’s analysis of variance for MS-AP and MS-A and one-way analysis of variance for MS-ML. Significant omnibus findings were followed by Bonferroni-adjusted pairwise comparisons using Welch *t*-tests for MS-AP and MS-A and pooled-variance *t*-tests for MS-ML. Omega-squared (ω^2^) was reported for omnibus age comparisons. Pearson’s correlation coefficient was used to examine relationships between age and morphometric parameters and among the morphometric parameters using participant-level bilateral mean values.

Participant-level shape was classified as bilateral trapezoidal, bilateral hourglass-shaped, or asymmetric. Associations of shape pattern with sex and age group were evaluated using the Fisher–Freeman–Halton exact test, with Cramér’s V reported for significant associations. The age-group analysis used a Monte Carlo estimate based on 1,000,000 samples because of sparse cell frequencies. Paired right–left shape distributions were compared using the exact McNemar test. Because pneumatization was uncommon, no inferential comparisons according to sex, side, or age group were performed; its prevalence was reported with an exact 95% binomial confidence interval. All statistical tests were two-sided, and *p* values below 0.05 were considered statistically significant. 

ChatGPT (OpenAI, San Francisco, CA, USA; GPT-5.6 Sol) was used during manuscript preparation to support drafting and improve English-language clarity and readability. All AI-assisted content was critically reviewed, revised, and approved by the authors, who assume full responsibility for the accuracy, integrity, and final content of the manuscript.

## 3. Results

### 3.1. Final Analytic Sample

**Figure 4:**
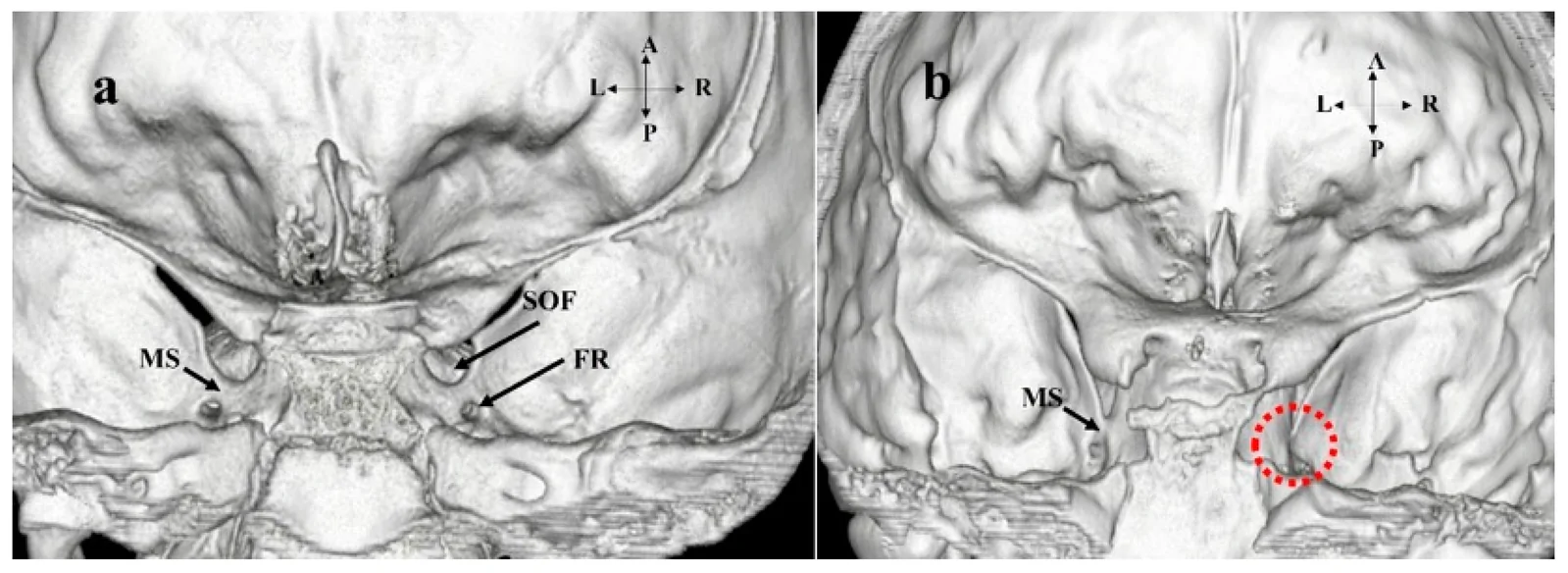
Three-dimensional computed tomography reconstructions demonstrating (**a**) bilateral maxillary struts (MS) between the foramen rotundum (FR) and superior orbital fissure (SOF) and (**b**) a rudimentary right-sided MS (red dashed circle). A, anterior; P, posterior; R, right; L, left.

One right-sided MS in a 17-year-old male participant was rudimentary (Figure 4). This participant was excluded from the primary complete-case morphometric, shape, and pneumatization analyses. Consequently, the primary bilateral complete-case analyses included 179 children (90 girls and 89 boys), 358 MSs (179 right and 179 left), and 179 complete bilateral pairs. 

### 3.2. Measurement Reliability and Observer Agreement

Interobserver reliability was evaluated using two-way random-effects, absolute-agreement, average-measures intraclass correlation coefficients [ICC(2,2)]. Interobserver reliability was excellent for MS-AP (ICC = 0.940, 95% confidence interval [CI]: 0.920–0.956), MS-A (ICC = 0.909, 95% CI: 0.878–0.932), and MS-ML (ICC = 0.936, 95% CI: 0.914–0.952; all *p* < 0.001). For the categorical assessments, right- and left-sided findings were combined at the participant level. MS shape was categorized as bilateral trapezoidal, bilateral hourglass-shaped, or asymmetric, whereas pneumatization was categorized as absent, unilateral, or bilateral. Agreement between the primary consensus classification and the blinded independent reassessment was observed for MS shape in 171 out of 179 participants (95.5%), yielding a Cohen’s kappa of 0.932 (95% CI: 0.882–0.974). For pneumatization, the two classifications agreed in 176 out of 179 participants (98.3%), with a Cohen’s kappa of 0.719 (95% CI: 0.279–1.000). The wide confidence interval for pneumatization reflected the very small number of positive findings (*n* = 4).

### 3.3. Sex- and Side-Related Morphometric Findings

Sex- and side-based comparisons are presented in Table 1. Levene’s tests indicated unequal variances between the sexes for MS-AP [F(1, 177) = 21.561, *p* < 0.001] and MS-A [F(1, 177) = 13.637, *p* < 0.001], but not for MS-ML [F(1, 177) = 0.025, *p* = 0.873], supporting the use of Welch’s tests for MS-AP and MS-A. Male participants had significantly higher values than female participants for MS-AP (mean difference: 0.891 mm; 95% CI: 0.492–1.289; *p* < 0.001; Hedges’ g = 0.662), MS-A (mean difference: 1.276 mm^2^; 95% CI: 0.342–2.211; *p* = 0.008; Hedges’ g = 0.403), and MS-ML (mean difference: 0.337 mm; 95% CI: 0.213–0.460; *p* < 0.001; Hedges’ g = 0.802). Paired-samples *t*-tests showed no significant right–left difference for MS-AP (mean difference: 0.106 mm; 95% CI: −0.098 to 0.311; *p* = 0.307; Cohen’s dz = 0.077), MS-A (mean difference: 0.302 mm^2^; 95% CI: −0.300 to 0.903; *p* = 0.324; Cohen’s dz = 0.074), or MS-ML (mean difference: −0.013 mm; 95% CI: −0.082 to 0.056; *p* = 0.714; Cohen’s dz = −0.027).

### 3.4. Age-Related Morphometric Findings

All three morphometric parameters differed significantly across the 18 age strata (Table 2). Across the 18 chronological age strata, Levene’s tests indicated unequal variances for MS-AP [F(17, 161) = 10.085, *p* < 0.001] and MS-A [F(17, 161) = 1.710, *p* = 0.046], but not for MS-ML [F(17, 161) = 1.333, *p* = 0.178]. Corresponding tests across the five prespecified chronological age groups were significant for MS-AP [F(4, 174) = 21.397, *p* < 0.001] and MS-A [F(4, 174) = 2.777, *p* = 0.029], but not for MS-ML [F(4, 174) = 1.852, *p* = 0.121]. Significant age-related differences were observed for MS-AP [F(17, 59.78) = 7.971, *p* < 0.001, ω^2^ = 0.355], MS-A [F(17, 59.75) = 6.354, *p* < 0.001, ω^2^ = 0.341], and MS-ML [F(17, 161) = 5.891, *p* < 0.001, ω^2^ = 0.317].

Comparisons across age groups demonstrated significant differences in MS-AP [F(4, 77.89) = 24.436, *p* < 0.001, ω^2^ = 0.330], MS-A [F(4, 80.57) = 20.342, *p* < 0.001, ω^2^ = 0.279], and MS-ML [F(4, 174) = 19.850, *p* < 0.001, ω^2^ = 0.296] (Table 3). Bonferroni-adjusted pairwise comparisons showed that for MS-AP and MS-A, Groups 1 and 2 did not differ significantly, Groups 3 and 4 did not differ significantly, Groups 1 and 2 had lower values than Groups 3 and 4, and Group 5 had higher values than all other groups. For MS-ML, no significant differences were found among Groups 1–3 or between Groups 3 and 4; Group 4 had higher values than Groups 1 and 2, and Group 5 had higher values than all other groups (all adjusted *p* < 0.05). The highest mean values were observed in Group 5 for MS-AP (5.35 mm), MS-A (15.31 mm^2^), and MS-ML (2.92 mm), indicating an overall tendency toward larger MS dimensions in the older age groups. 

### 3.5. Correlations Between Age and Morphometric Parameters

Age showed moderate positive correlations with MS-AP (r = 0.573, *p* < 0.001), MS-A (r = 0.561, *p* < 0.001), and MS-ML (r = 0.572, *p* < 0.001). Significant moderate positive correlations were also observed between MS-AP and MS-A (r = 0.582, *p* < 0.001), MS-AP and MS-ML (r = 0.535, *p* < 0.001), and MS-A and MS-ML (r = 0.511, *p* < 0.001).

### 3.6. Age- and Sex-Related Regression Models

**Figure 5:**
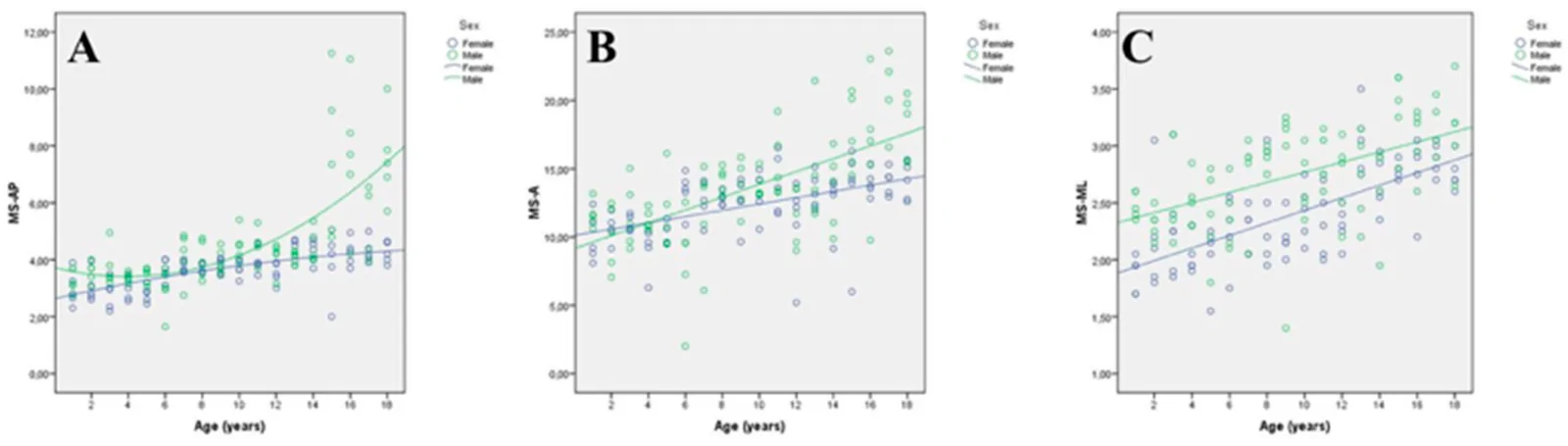
Sex-specific age-related regression models for maxillary strut morphometric parameters. Open circles represent participant-level bilateral mean observations, and the lines represent the fitted sex-specific regression trajectories. (**A**) MS-AP, modeled using quadratic functions with sex-specific curves; (**B**) MS-A, modeled using linear functions with sex-specific slopes; and (**C**) MS-ML, modeled using linear functions with parallel slopes for females and males. MS-AP, maxillary strut anteroposterior diameter; MS-A, maxillary strut area; MS-ML, maxillary strut mediolateral diameter.

Regression analysis demonstrated that the age-related patterns of MS-AP and MS-A differed by sex (Table 4 and Figure 5). For MS-AP, the fitted equations were MS-AP = 2.631 + 0.145 × age − 0.00291 × age^2^ for females and MS-AP = 3.725 − 0.161 × age + 0.0204 × age^2^ for males (adjusted R^2^ = 0.542; age-by-sex interaction, *p* < 0.001). For MS-A, the fitted equations were MS-A = 10.111 + 0.230 × age for females and MS-A = 9.153 + 0.469 × age for males (adjusted R^2^ = 0.383; age-by-sex interaction, *p* = 0.001). MS-ML increased linearly with age, with the equations MS-ML = 1.932 + 0.0499 × age for females and MS-ML = 2.273 + 0.0499 × age for males (adjusted R^2^ = 0.465; overall model, *p* < 0.001). The age-by-sex interaction was not significant for MS-ML (*p* = 0.257), indicating parallel age-related slopes in females and males. Age was expressed in years in all equations.

Residual diagnostics identified heteroscedasticity in the MS-AP (Koenker–Breusch–Pagan Lagrange multiplier [LM] = 33.367, df = 5, *p* < 0.001) and MS-A (LM = 10.717, df = 3, *p* = 0.013) models, but not in the MS-ML model (LM = 2.493, df = 2, *p* = 0.288). Residual Q–Q plots and Shapiro–Wilk tests indicated tail departures from normality for MS-AP (W = 0.839, *p* < 0.001), MS-A (W = 0.955, *p* < 0.001), and to a lesser extent, MS-ML (W = 0.980, *p* = 0.010). Four MS-AP, three MS-A, and three MS-ML observations had absolute externally studentized residuals greater than 3; however, the maximum Cook’s distances were 0.150, 0.065, and 0.077, respectively, and no observation had a Cook’s distance greater than 1. Diagnostic refitting after excluding these observations did not change the inferential conclusions: the joint age-by-sex interaction remained significant for MS-AP (*p* < 0.001), the age-by-sex interaction remained significant for MS-A (*p* = 0.004), and the interaction remained nonsignificant for MS-ML (*p* = 0.149).

### 3.7. Sensitivity Analysis

The sensitivity analysis (*n* = 180) yielded the same inferential conclusions as the primary analysis. Sex-related differences remained significant for MS-AP (*p* < 0.001), MS-A (*p* = 0.004), and MS-ML (*p* < 0.001). Differences across both the 18 chronological age strata and the five age groups remained significant for all three parameters (all *p* < 0.001), and the pattern of significant post hoc comparisons was unchanged. All age–morphometry and inter-parameter correlations remained significant (r = 0.432–0.575; all *p* < 0.001). In the regression analyses, the age-by-sex interactions remained significant for MS-AP and MS-A (both *p* < 0.001), whereas the interaction for MS-ML remained nonsignificant (*p* = 0.146).

### 3.8. Distribution of Maxillary Strut Shape

Among the 179 participants included in the shape analysis, 73 (40.8%) had bilateral trapezoidal MSs, 54 (30.2%) had bilateral hourglass-shaped MSs, and 52 (29.1%) had an asymmetric configuration. MS shape pattern was significantly associated with sex (*p* = 0.003; Cramér’s V = 0.253). An asymmetric configuration was more frequent in males than in females (36/89, 40.4% vs. 16/90, 17.8%), whereas bilateral trapezoidal (32/89, 36.0% vs. 41/90, 45.6%) and bilateral hourglass-shaped configurations (21/89, 23.6% vs. 33/90, 36.7%) were more frequent in females. Across pediatric age Groups 1–5, bilateral trapezoidal configurations were observed in 7 (35.0%), 12 (40.0%), 15 (37.5%), 13 (32.5%), and 26 (53.1%) participants, respectively. The corresponding frequencies of bilateral hourglass-shaped configurations were 6 (30.0%), 8 (26.7%), 12 (30.0%), 12 (30.0%), and 16 (32.7%), while asymmetric configurations were observed in 7 (35.0%), 10 (33.3%), 13 (32.5%), 15 (37.5%), and 7 (14.3%) participants, respectively. MS shape pattern was not significantly associated with pediatric age group (*p* = 0.359). In the side-based paired analysis, the trapezoidal type was identified in 92 right-sided (51.4%) and 106 left-sided MSs (59.2%), whereas the hourglass-shaped type was identified in 87 right-sided (48.6%) and 73 left-sided MSs (40.8%). The right–left difference in shape distribution was not statistically significant (exact McNemar test, *p* = 0.070).

### 3.9. Frequency of Maxillary Strut Pneumatization

Pneumatization was identified in 4 out of 179 participants (2.2%; exact 95% CI: 0.6–5.6%). All cases were unilateral, involving the right side in three participants and the left side in one participant; no bilateral pneumatization was observed. 

## 4. Discussion

Understanding skull-base development is important for interpreting the pediatric anatomy of the MS, which arises from the greater wing of the sphenoid. The cranial base begins to develop during fetal life and continues to mature after birth [13,14,15,16]. Most of it forms through endochondral ossification of cartilaginous precursors derived from neural crest cells and mesoderm, whereas smaller regions develop through intramembranous ossification [14,16]. The sphenoid bone has a particularly complex developmental pattern involving the presphenoid, postsphenoid, orbitosphenoid, and alisphenoid ossification centers [14,15]. Of particular relevance to the MS, the alisphenoid center appears at approximately the 15th gestational week and contributes to the medial portion of the greater wing, whereas the lateral portion of the greater wing and the lateral pterygoid plate also receive intramembranous contributions [14,15]. As cranial-base maturation continues after birth, the MS may undergo further morphological changes throughout childhood and adolescence. This prolonged developmental process provides an anatomical basis for the age-related variations observed in pediatric MS morphology [13].

The MS is an important surgical landmark in endoscopic skull base surgery because it marks the boundary between the SOF and the FR, thereby helping the surgeon navigate the anatomical transition zone between the pterygopalatine fossa and the middle cranial fossa [1]. From the endonasal perspective, the MS forms the inferior border of the medial aspect of the SOF, and together with the optic strut, facilitates surgical orientation toward the orbital apex and cavernous sinus; its relationship with adjacent neurovascular structures further enhances its surgical value [3]. The MS is also functionally relevant for V2-oriented surgery, because it corresponds to the intracanalicular segment of V2, with an average anteroposterior length of approximately 4.4 mm, while the segment posterior to the strut defines an additional interdural corridor of about 9 mm that can be used for controlled mobilization or resection of infiltrated V2 without violating the middle fossa dura [2]. In clinical practice, drilling the MS can expand access to the middle fossa and facilitate the resection of lesions arising from or extending through the FR/SOF region, including tumors with perineural spread along V2 [1,2]. More recent studies have further shown that the MS serves as the inferior limit of SOF decompression in both endonasal and transorbital approaches, making it a practical boundary for targeted decompression in traumatic SOF syndrome [4]. In transrotundum middle fossa surgery, V2 transposition combined with resection of most of the MS has been associated with a marked increase in lateral middle fossa exposure, from about 50% to 95%, underscoring its importance not only as a landmark, but also as a gateway for expanding minimally invasive skull base corridors [5]. A subset of pathologies in pediatric patients may require surgical management in areas anatomically related to the SOF and/or the FR. For instance, Cohen and Couldwell [17] reported successful surgical management of a 14-year-old patient with a meningioma involving the SOF and anterior cavernous sinus via a lateral orbitotomy approach; the lesion had presented with ocular pain, strabismus, and ptosis. Given that the current literature on the MS is largely derived from adult populations [1,2,3] and that related pathologies may also occur in children [6,17], the present study provides descriptive pediatric anatomical data on age- and sex-related MS development; its potential relevance to preoperative assessment and surgical planning in the SOF–FR region requires further clinical validation. Recent pediatric research has demonstrated that CT-based three-dimensional virtual-reality simulation can be used to evaluate the feasibility and vascular safety of patient-specific trajectories to the pterygopalatine fossa [18]. Incorporating the MS, FR, SOF, and adjacent neurovascular structures into similar models could allow for future investigation of how individual MS anatomy influences skull-base trajectory planning. However, the present study did not include trajectory simulation or surgical validation; therefore, this potential application remains a subject for future research.

Morphometric studies specifically focusing on the MS remain notably limited in the literature. Grewal et al. [1] assessed the morphometry of the MS in 100 dry skulls using both direct caliper-based measurements and CT/BrainLab analysis. Using caliper measurements, the mean values for MS-AP were 4.18 ± 0.15 mm on the right and 3.90 ± 0.14 mm on the left. Using CT/BrainLab analysis, the mean MS-A was 15.25 ± 0.48 mm^2^, and the mean strut thickness was 4.43 ± 0.10 mm [1]. In contrast, Abhinav et al. [2] studied five silicone-injected human head specimens bilaterally (10 sides) using an endoscopic endonasal transpterygoid approach and reported that the intracanalicular segment of V2, corresponding to MS-AP, measured 4.4 ± 0.5 mm. In our pediatric series, the overall mean values were 4.11 ± 1.41 mm for MS-AP, 12.93 ± 3.21 mm^2^ for MS-A, and 2.57 ± 0.45 mm for MS-ML. When compared with the limited literature, our overall pediatric MS-AP values appear broadly comparable to the adult-based values reported by Grewal et al. [1] and slightly lower than those reported by Abhinav et al. [2], whereas our overall MS-A value is lower than that reported by Grewal et al. [1]. MS-AP, MS-A, and MS-ML differed significantly across both chronological age strata and age groups, with generally smaller dimensions in the younger groups and the highest mean values in Group 5 (14–18 years). Male participants had higher values than female participants for all three parameters, whereas no significant right–left differences were observed. Regression analysis showed sex-specific quadratic trajectories for MS-AP and different linear age-related slopes for MS-A, whereas MS-ML increased linearly with parallel slopes in females and males. Age-related MS development appeared to vary according to both morphometric parameter and sex. These regression equations should be interpreted as descriptive models of age- and sex-related trends within this cohort rather than as tools for predicting individual MS dimensions; external validation is required before any clinical predictive use. Comparisons with adult studies should nevertheless be interpreted cautiously because of differences in study populations, imaging methods, and measurement techniques. Further pediatric clinical and surgical studies may be useful to better evaluate the potential clinical applicability of the present anatomical findings.

Anatomical data specifically addressing variations of the MS remain very limited in the literature. In one of the few studies focusing directly on the MS, Grewal et al. [1] described it as a trapezoidal bony structure and reported that it was present bilaterally in all skulls examined, supporting the view that the MS is usually a constant anatomical landmark. In our series, a rudimentary right-sided MS was identified in a 17-year-old male subject, indicating that this variation is rare. At the participant level, bilateral trapezoidal, bilateral hourglass-shaped, and asymmetric configurations were observed in 40.8%, 30.2%, and 29.1% of participants, respectively. MS shape pattern was significantly associated with sex, with asymmetric configurations occurring more frequently in males. No significant association was found with age group, and the paired right–left shape distributions did not differ significantly. Data on MS pneumatization appear to be particularly scarce in the existing literature. Pneumatization was identified in 4 out of 179 participants. All four findings were unilateral, involving the right side in three participants and the left side in one participant. Because pneumatization was identified in only four participants, inferential comparisons according to sex, side, or age group were not performed. Taken together, these findings indicate that rudimentary morphology and pneumatization are uncommon, whereas MS shape demonstrates appreciable interindividual variation and a sex-related distribution. 

This study has several limitations. First, the moderate sample size limited precision, particularly for rare findings such as rudimentary morphology and pneumatization. Second, the deliberately age- and sex-balanced sampling design did not reflect the age and sex distribution of the source population, and the absence of a screening log prevented reconstruction of the exact numbers of records screened, eligible, and excluded. Furthermore, the cohort comprised pediatric patients who underwent CT for clinical indications rather than healthy volunteers, introducing potential selection bias and limiting generalizability, although CT imaging of healthy children solely for research would not be ethically justified. Third, the retrospective design restricted the availability of anthropometric and pubertal-development data. Fourth, measurements were obtained from sectional CT images rather than dedicated three-dimensional volumetric analyses; the observers were not blinded to age or sex, and the absence of repeated measurement sessions precluded the assessment of intraobserver reliability. Finally, the regression models were not internally or externally validated, and the study did not evaluate surgical exposure, drilling safety, operative outcomes, or clinically applicable thresholds. Accordingly, the findings should be interpreted as cohort-specific descriptive anatomical data requiring validation in larger and more representative populations.

## 5. Conclusions

The present study demonstrates that the MS undergoes measurable age- and sex-related morphological changes throughout childhood and adolescence. Morphometric dimensions generally increased with age, with the highest values observed in the 14–18-year age group, and were significantly higher in males, whereas no significant right–left differences were identified. MS shape showed substantial interindividual variation and a sex-related distribution, while pneumatization was uncommon and exclusively unilateral. These pediatric CT-based data may provide a useful anatomical reference for future radiologic, developmental, and surgical studies of the skull base, particularly those involving the region between the FR and the SOF.

## Figures and Tables

**Table 1:** Sex- and side-based comparison of maxillary strut morphometric measurements in children.

Parameters	Male (*n* = 89)	Female (*n* = 90)	*p* Value	Right (*n* = 179)	Left (*n* = 179)	*p* Value
MS-AP (mm)	4.56 ± 1.77	3.67 ± 0.69	<0.001	4.16 ± 1.51	4.06 ± 1.63	0.307
MS-A (mm^2^)	13.57 ± 3.85	12.30 ± 2.26	0.008	13.08 ± 3.81	12.78 ± 3.79	0.324
MS-ML (mm)	2.74 ± 0.43	2.41 ± 0.41	<0.001	2.57 ± 0.50	2.58 ± 0.51	0.714

Data are presented as mean ± standard deviation. Sex-based comparisons were performed at the participant level using the mean of the right- and left-sided measurements. MS-AP and MS-A were compared between males and females using Welch’s *t*-test because of unequal variances, whereas MS-ML was compared using the independent-samples *t*-test. Right- and left-sided measurements were compared using the paired-samples *t*-test based on 179 bilateral pairs. All reported *p* values are two-sided. Abbreviations: *n*, number of participants; MS-AP, maxillary strut anteroposterior diameter; MS-A, maxillary strut area; MS-ML, maxillary strut mediolateral diameter.

**Table 2:** Age-specific distribution of maxillary strut morphometric measurements in children.

Age (Years)	*n*	MS-AP (mm)	MS-A (mm^2^)	MS-ML (mm)
1	10	3.13 ± 0.52	10.77 ± 1.62	2.18 ± 0.35
2	10	3.30 ± 0.52	10.31 ± 1.75	2.26 ± 0.35
3	10	3.27 ± 0.77	11.28 ± 1.71	2.38 ± 0.43
4	10	3.19 ± 0.35	10.21 ± 1.65	2.27 ± 0.31
5	10	3.12 ± 0.43	10.89 ± 2.08	2.21 ± 0.37
6	10	3.31 ± 0.70	10.75 ± 3.89	2.33 ± 0.29
7	10	3.94 ± 0.62	12.32 ± 2.63	2.51 ± 0.39
8	10	3.88 ± 0.48	13.48 ± 1.01	2.63 ± 0.42
9	10	3.87 ± 0.35	13.16 ± 1.82	2.50 ± 0.63
10	10	4.10 ± 0.60	13.44 ± 1.35	2.54 ± 0.28
11	10	4.22 ± 0.57	14.63 ± 2.37	2.59 ± 0.38
12	10	3.82 ± 0.53	11.22 ± 2.69	2.51 ± 0.34
13	10	4.23 ± 0.32	14.00 ± 2.85	2.88 ± 0.37
14	10	4.37 ± 0.49	13.05 ± 2.38	2.66 ± 0.32
15	10	5.72 ± 2.77	15.37 ± 4.03	3.06 ± 0.36
16	10	5.97 ± 2.46	15.27 ± 3.54	2.92 ± 0.33
17	9	4.73 ± 1.00	16.96 ± 3.97	3.02 ± 0.24
18	10	5.91 ± 2.06	16.06 ± 2.80	2.96 ± 0.34
9.5 ± 5.2	179	4.11 ± 1.41	12.93 ± 3.21	2.57 ± 0.45
*p* value		<0.001	<0.001	<0.001

Data are presented as mean ± standard deviation. Analyses were performed at the participant level using the mean of the right- and left-sided measurements. Overall differences across the 18 chronological age strata were evaluated using Welch’s analysis of variance for MS-AP and MS-A and one-way analysis of variance for MS-ML. The reported *p* values represent omnibus comparisons across the 18 age strata; pairwise post hoc comparisons were not performed. The 17-year age stratum included nine participants because one participant with a rudimentary unilateral maxillary strut was excluded from the morphometric analyses. All reported *p* values are two-sided. Abbreviations: *n*, number of participants; MS-AP, maxillary strut anteroposterior diameter; MS-A, maxillary strut area; MS-ML, maxillary strut mediolateral diameter.

**Table 3:** Comparison of maxillary strut morphometric measurements according to pediatric age groups.

Parameters	Group 1	Group 2	Group 3	Group 4	Group 5	*p* Value
MS-AP (mm)	3.22 ± 0.51 ^a^	3.19 ± 0.53 ^a^	3.75 ± 0.59 ^b^	4.09 ± 0.53 ^b^	5.35 ± 2.01 ^c^	<0.001
MS-A (mm^2^)	10.54 ± 1.66 ^a^	10.79 ± 1.81 ^a^	12.43 ± 2.69 ^b^	13.32 ± 2.64 ^b^	15.31 ± 3.50 ^c^	<0.001
MS-ML (mm)	2.22 ± 0.34 ^a^	2.28 ± 0.37 ^a^	2.49 ± 0.45 ^a,b^	2.63 ± 0.37 ^b^	2.92 ± 0.34 ^c^	<0.001

Data are presented as mean ± standard deviation. Group 1 included 20 children aged 1–2 years, Group 2 included 30 children aged 3–5 years, Group 3 included 40 children aged 6–9 years, Group 4 included 40 children aged 10–13 years, and Group 5 included 49 children aged 14–18 years. Analyses were performed at the participant level using the mean of the right- and left-sided measurements. Group differences in MS-AP and MS-A were evaluated using Welch’s analysis of variance, whereas MS-ML was evaluated using one-way analysis of variance. Significant omnibus differences were further examined using Bonferroni-adjusted pairwise comparisons. Pairwise Welch *t*-tests were used for MS-AP and MS-A, and pooled-variance pairwise *t*-tests were used for MS-ML. Values sharing at least one superscript letter are not significantly different (Bonferroni-adjusted *p* ≥ 0.05). Abbreviations: MS-AP, maxillary strut anteroposterior diameter; MS-A, maxillary strut area; MS-ML, maxillary strut mediolateral diameter.

**Table 4:** Regression coefficients for age- and sex-related maxillary strut morphometric models.

Outcome	Model Term	β	SE	95% CI	*p* Value
MS-AP	Intercept	2.631	0.197	2.242 to 3.020	<0.001
MS-AP	Age (years)	0.145	0.042	0.062 to 0.228	0.001
MS-AP	Age^2^	−0.00291	0.00200	−0.00685 to 0.00104	0.148
MS-AP	Male sex	1.094	0.309	0.483 to 1.704	0.001
MS-AP	Age × male sex	−0.306	0.089	−0.481 to −0.131	0.001
MS-AP	Age^2^ × male sex	0.02330	0.00542	0.01260 to 0.03400	<0.001
MS-A	Intercept	10.111	0.349	9.423 to 10.799	<0.001
MS-A	Age (years)	0.230	0.035	0.162 to 0.298	<0.001
MS-A	Male sex	−0.957	0.688	−2.316 to 0.401	0.166
MS-A	Age × male sex	0.239	0.072	0.098 to 0.380	0.001
MS-ML	Intercept	1.932	0.057	1.819 to 2.044	<0.001
MS-ML	Age (years)	0.0499	0.0048	0.0405 to 0.0593	<0.001
MS-ML	Male sex	0.341	0.049	0.244 to 0.438	<0.001

Female sex was the reference category (male = 1). Coefficients are reported on the chronological-age scale used in the fitted equations. HC3 heteroscedasticity-consistent standard errors were used for MS-AP and MS-A; conventional ordinary least-squares standard errors were used for MS-ML. The linear and quadratic age-by-sex terms in the MS-AP model were jointly significant (HC3-robust Wald F(2, 173) = 11.298, *p* < 0.001). In an expanded MS-ML model, the age-by-sex interaction was not significant (β = −0.0108, SE = 0.0095, 95% CI: −0.0296 to 0.0079, *p* = 0.257) and was therefore not retained. β, regression coefficient; CI, confidence interval; SE, standard error; MS-AP, maxillary strut anteroposterior diameter; MS-A, maxillary strut area; MS-ML, maxillary strut mediolateral diameter.

## Data Availability

Available from the corresponding author on reasonable request.

## Abbreviations

ANOVA	Analysis of Variance
CT	Computed Tomography
FR	Foramen Rotundum
ICC	Intraclass Correlation Coefficient
MS	Maxillary Strut
MS-A	Maxillary Strut Area
MS-AP	Maxillary Strut Anteroposterior Diameter
MS-ML	Maxillary Strut Mediolateral Diameter
R^2^	Coefficient of Determination
SOF	Superior Orbital Fissure
SPSS	Statistical Package for the Social Sciences
TABED	Medical Research Scientific and Ethical Evaluation Board (Türkiye)
3D	Three-Dimensional
CI	Confidence Interval
HC3	Heteroscedasticity-Consistent Standard Error Estimator, Type 3
HU	Hounsfield Unit
LM	Lagrange Multiplier
Q–Q	Quantile–Quantile
SE	Standard Error
V2	Maxillary Division of the Trigeminal Nerve
